# Psychosocial Needs of Gynecological Cancer Survivors: Mixed Methods Study

**DOI:** 10.2196/37757

**Published:** 2022-09-20

**Authors:** Elizabeth J Adams, David Tallman, Marcy L Haynam, Larissa Nekhlyudov, Maryam B Lustberg

**Affiliations:** 1 Feinberg School of Medicine Northwestern University Chicago, IL United States; 2 Wexner Medical Center The Ohio State University Columbus, OH United States; 3 Brigham and Women’s Hospital Harvard Medical School Boston, MA United States; 4 Yale Cancer Center School of Medicine Yale University New Haven, CT United States

**Keywords:** mixed methods, quantitative, qualitative, cancer survivorship, gynecological cancer, uterine cancer, ovarian cancer, cancer informatics, patient discussion, social media

## Abstract

**Background:**

Internet and social media platforms offer insights into the lived experiences of survivors of cancer and their caregivers; however, the volume of narrative data available is often cumbersome for thorough analysis. Survivors of gynecological cancer have unique needs, such as those related to a genetic predisposition to future cancers, impact of cancer on sexual health, the advanced stage at which many are diagnosed, and the influx of new therapeutic approaches.

**Objective:**

This study aimed to present a unique methodology to leverage large amounts of data from internet-based platforms for mixed methods analysis. We analyzed discussion board posts made by survivors of gynecological cancer on the American Cancer Society website with a particular interest in evaluating the psychosocial aspects of survivorship.

**Methods:**

All posts from the ovarian, uterine, and gynecological cancers (other than ovarian and uterine) discussion boards on the American Cancer Society Cancer Survivors Network were included. Posts were web scraped using Python and organized by psychosocial themes described in the Quality of Cancer Survivorship Care Framework. Keywords related to each theme were generated and verified. Keywords identified posts related to the predetermined psychosocial themes. Quantitative analysis was completed using Python and R Foundation for Statistical Computing packages. Qualitative analysis was completed on a subset of posts as a proof of concept. Themes discovered through latent Dirichlet allocation (LDA), an unsupervised topic modeling technique, were assessed and compared with the predetermined themes of interest.

**Results:**

A total of 125,498 posts made by 6436 survivors of gynecological cancer and caregivers between July 2000 and February 2020 were evaluated. Of the 125,489 posts, 23,458 (18.69%) were related to the psychosocial experience of cancer and were included in the mixed methods psychosocial analysis. Quantitative analysis (23,458 posts) revealed that survivors across all gynecological cancer discussion boards most frequently discussed the role of friends and family in care, as well as fatigue, the effect of cancer on interpersonal relationships, and health insurance status. Words related to psychosocial aspects of survivorship most often used in posts included “family,” “hope,” and “help.” Qualitative analysis (20 of the 23,458 posts) similarly demonstrated that survivors frequently discussed coping strategies, distress and worry, the role of family and caregivers in their cancer care, and the toll of managing financial and insurance concerns. Using LDA, we discovered 8 themes, none of which were directly related to psychosocial aspects of survivorship. Of the 56 keywords identified by LDA, 2 (4%), “sleep” and “work,” were included in the keyword list that we independently devised.

**Conclusions:**

Web-based discussion platforms offer a great opportunity to learn about patient experiences of survivorship. Our novel methodology expedites the quantitative and qualitative analyses of such robust data, which may be used for additional patient populations.

## Introduction

### Background

Gynecological cancer is a broad disease category that includes cervical, uterine, ovarian, vaginal, and vulvar cancers, which are distinct in their presentation, pathology, treatment, prognosis, and survivorship trajectories. It is estimated that there were approximately 116,760 new cases and 34,080 new deaths from gynecological cancers in 2021 [1]. The 5-year overall survival rates for uterine and ovarian cancer are now 81% and 48%, respectively [1]. Although there is vast room for improvement in these survival rates through earlier detection and improved treatment, there has been notable progress in the management and survival of those with gynecological cancer over time. Thus, there is a larger population of survivors of gynecological cancer now than ever before. With this growing population of survivors of gynecological cancer, there is a paramount need to investigate methods to both assess and implement survivorship-focused care among survivors diagnosed and living with gynecological cancer.

Cancer survivorship was first described in 2006 in the landmark publication “From Cancer Patient to Cancer Survivor: Lost in Transition” [2], which outlined the gap in care between active cancer treatment and life after completion of active cancer treatment. Much work has been conducted in survivorship since 2006, with an emphasis on survivorship beginning on the day of the cancer diagnosis. This is reflected in the National Cancer Institute’s definition of survivorship: the “health and well-being of a person with cancer from the time of diagnosis until the end of life” [3]. Thus, survivorship care is focused on every aspect of health and wellness that is not directly related to treating the cancer itself.

The National Comprehensive Cancer Network provides guidelines on the delivery of survivorship care and outlines 7 aspects of survivorship care, including preventing new and recurrent cancers, preventing late effects of cancer and treatment, surveillance for return of cancer, screening for new cancers, assessing and treating late effects of cancer and treatment, coordinating care among providers, and planning for ongoing survivorship care [4]. Additional guidelines, such as those by the American Cancer Society (ACS), the American Society of Clinical Oncology, and Cancer Care Ontario, assert similar components as essential aspects of survivorship care [5-7]. The Quality of Cancer Survivorship Care Framework defines domains of survivorship care through an iterative review of survivorship care guidelines that was bolstered with key expert interviews [8]. All guidelines emphasize the importance of considering the psychosocial aspects of survivorship, which the National Cancer Institute defines as the “mental, emotional, social, and spiritual effects of a disease” with the psychosocial effects of cancer, including “changes in how a patient thinks, their feelings, moods, beliefs, ways of coping, and relationships with family, friends, and co-workers” [9]. The psychosocial effects of cancer and cancer treatment are an important area for exploration as they are unique to each cancer type, cancer treatment, and individual patient.

### Digitalizing Survivorship

As survivorship is a relatively new component of oncologic care, the field continues to develop new interventions and modify existing initiatives to best meet the needs of survivors. Learning directly from the voices of survivors is an essential step to inform these survivorship services. Web-based discussion boards and social media platforms have become tools for researchers and clinicians to harness publicly available discussions on cancer survivorship, offering extremely large quantities of candid and spontaneous thoughts and opinions from survivors [10]. Gao et al [11] recently demonstrated the utility of investigating posts made on Instagram related to head and neck cancer, where they reported that most of the posts made by patients were focused on appointments, treatment, and side effects. Similar analyses have been conducted on Twitter [12] and Facebook [13] posts for additional cancer types. Discussion-based forums, including Facebook groups [14] and those on the ACS website [15], have also been analyzed by researchers to acquire a better understanding of survivorship from the perspective of survivors of cancer. Although these prior studies demonstrated the utility of analyzing posts and discussions among survivors, each was conducted with manual thematic analysis. The feasibility of organizing and analyzing text-based posts and discussions by hand has an upper limit, which undermines one of the greatest strengths of exploring such posts—the volume of data available. To overcome this, other researchers have used automated technology such as unsupervised topic modeling to determine the most prevalent themes in web-based and social media posts [16-18]. However, this process does not necessarily identify themes that are of importance to the researcher, such as when the themes are related to particular aspects of survivorship care, and does not detect predetermined themes of interest.

### Objective

The ACS Cancer Survivors Network has discussion boards specific to types of cancer, where survivors and caregivers can interact through posts. To demonstrate a novel methodology to scan through an extensive compilation of these posts and thematically organize them, we analyzed the ACS gynecological cancer discussion boards, including the ovarian, uterine, and gynecological cancers (other than ovarian and uterine) discussion boards. Our methodology automated the analysis of >125,000 discussion board posts using the previously described Quality of Cancer Survivorship Care Framework, with a specific focus on the themes within the framework’s *Surveillance and Management of Psychosocial Effects Domain* [8]. Our goal was to demonstrate the utility of this novel methodology to expedite the guided quantitative and qualitative analysis of a robust amount of discussion-based data from survivors of gynecological cancer and caregivers based on predetermined themes of interest, such as those in the Quality of Cancer Survivorship Care Framework.

## Methods

### Study Design

This study uses a mixed methods approach, specifically a sequential explanatory design [19]. Sequential explanatory studies involve two phases in the analysis. First, a quantitative analysis is conducted, which informs a second, qualitative analysis phase [19,20]. The information from the first quantitative analysis and second qualitative analysis is combined using integration strategies. Per the standard sequential explanatory study design, there are 2 stages to data integration, with the first taking place after the conclusion of quantitative analysis. This was completed by determining which themes were most prevalent in the data set, which allowed us to connect the quantitative component of the study to the qualitative component. The second stage of data integration took place after the completion of qualitative analysis, during which we compared the quantitative and qualitative findings to share the reported findings [19,20]. Viewing both the findings from the quantitative analysis and those from the qualitative analysis as contributing equally to the results but in different ways is referred to as complementary stance integration [21]. To implement this, we completed the following iterative process that will be detailed in the paragraphs that follow: identify predetermined themes of interest, web scrape, develop keywords to capture themes in the web-based text, verify the validity and reliability of the keywords, apply the keywords to the data set, and analyze the data output.

This process was developed after an initial exploration of topic modeling, specifically latent Dirichlet allocation (LDA). Topic modeling is an unsupervised machine learning approach to determine patterns of related words in large quantities of text, thus independently discovering themes that the program determines to be of significance based on probability [22]. The Python packages *gensim* and *LDAvis* were used to facilitate theme discovery. To determine the best number of topics for our data set, the metric of coherence (C_V_) was used [23]. Models of varying numbers of topics ranging from 2 to 40 were developed in increments of 2, and the highest C_V_ score of 0.52 was observed with 8 themes. A relevance metric in the LDAvis package was used to evaluate the most relevant keywords rather than just the most prevalent as there was a high frequency of particular words (eg, “cancer”) based on the nature of the discussion board data. On the basis of correlation with human interpretability, the value of λ was set to 0.6 [24]. Consensus was reached among the study team on the semantic meaning of the 8 topics. These topic modeling–generated themes were compared with those in the Quality of Cancer Survivorship Care Framework [8]. A second attempt at LDA was used to generate a model with 16 themes, which had the second highest C_V_ score, as a means of more closely mimicking the number of psychosocial themes in the Quality of Cancer Survivorship Care Framework to obtain topics with more narrow themes.

### Predetermined Themes of Interest

The Quality of Cancer Survivorship Care Framework [8] has multiple quality domains, including the *Surveillance and Management of Psychosocial Effects Domain*; *Surveillance, and Management of Physical Effects* *Domain*; and *Care Coordination Domain*, among others*.* In this analysis, we focused on the *Surveillance and Management of Psychosocial Effects Domain*. Each domain contains several proposed quality indicators to assess different aspects of survivorship care; for example, within the *Surveillance and Management of Psychosocial Effects Domain*, the proposed quality indicators to assess cancer survivorship care include “fatigue” and “stress,” as well as “financial toxicity” and “fertility.” Although these indicators may be used to assess the quality of survivorship care, we felt that they encapsulated all of the relevant themes of interest that survivors of gynecological cancer may discuss on the web on the ACS discussion boards. Therefore, for the purposes of our study, we will be referring to the indicators listed in the framework’s *Surveillance and Management of Psychosocial Effects Domain* as “themes.” We use these themes to demonstrate the utility of our novel methodology by detecting the presence of these themes in communications between survivors of gynecological cancer and caregivers.

### Web Scraping

All 125,498 posts from the ovarian, uterine, and gynecological cancers (other than ovarian and uterine) ACS discussion boards were evaluated. The posts included in the analysis were created from July 21, 2000, through February 24, 2020, the date when web scraping was completed. Posts were either responses that were added to an existing conversation or the “conversation-starters” on the discussion board. Web scraping, or simply “scraping,” is a technique used to extract the content of interest from web-based platforms so that it can be analyzed using computer software, essentially “downloading” it in a way that can be used by researchers. Python is a computer programming language that automates specific actions performed on the computer, such as the process of web scraping. By creating a custom Python script, the process of scraping >125,000 posts was automated. Python has multiple packages that allow for the software to perform different actions. In our analysis, we specifically used the Python packages *urllib* and *Beautiful Soup* 4 to navigate and extract the text from discussion posts on the ACS discussion boards. First, *urllib* was used to interface with the ACS website and scrape the web page. Next, *Beautiful Soup* 4 was used to parse the HTML code obtained from the ACS web pages. Together, this allowed us to extract the submitted text contained in a post, the date of the post, and the username of the poster. After the posts were scraped from the ovarian, uterine, and gynecological cancers (other than ovarian and uterine) discussion boards, they were saved to a CSV data file that was used for further downstream analyses.

### Capturing Themes of Interest Using Keywords

We devised a list of “keywords” that captured each of the predetermined themes from the Quality of Cancer Survivorship Care Framework’s *Surveillance and Management of Psychosocial Effects Domain* (Textbox 1). Every theme had multiple keywords that were created from synonyms, related phrases, and related words. The keyword list used truncated, root words to capture all variations of a given keyword; for example, 1 theme in the *Surveillance and Management of Psychosocial Effects Domain* is “underemployment, unemployment, return to work.” One of the numerous keywords to capture this theme is based on the root word “unemploy.” By using the root word “unemploy,” the methodology captures all variations of it, including “unemployment,” “unemployed,” and “unemployable.” This allows us to capture all instances when survivors may have discussed topics related to the base word “unemploy.”

The purpose of the keyword list was to be able to determine which ACS posts discussed any of the predetermined themes of interest. Our methodology scans through data, detecting when the keywords were used. The use of a particular theme’s keywords indicated to us that the ACS post discussed that particular theme. The software noted how many times the keywords were present from every single predetermined theme across every single evaluated ACS post. Thus, each ACS post was assigned a “theme score” for every theme. This allowed us to determine which themes were most prevalent in a given post without reading it first based on the number of times that theme’s keywords appeared in it. Simultaneously, the theme score allowed us to immediately identify which ACS posts discussed specific themes of interest. This expedited the process as we were able to quickly pull up all of the posts relevant to particular themes, thus expediting additional review and qualitative analysis.

**Keyword list.**

**Surveillance and Management of Psychosocial Effects themes and keywords**

**Psychological**

“Psychological” and “psychology”

**Fatigue**

“Fatigue,” “tired,” “tiring,” “fatiguing,” “exhaust,” “nap,” and “rest”

**Stress**

“Stress” and “stressed”

**Posttraumatic stress**

“Posttraumatic stress,” “trauma,” and “traumatized”

**Posttraumatic growth**

“Posttraumatic growth,” “trauma,” and “traumatized”

**Distress**

“Distress,” “depression,” “depressed,” “feel down,” “feeling down,” “sad,” “sadness,” “tear,” “cry,” “upset,” “heartbroken,” “heartbreaking,” “wrench,” “guilt,” and “cried”

**Anxiety**

“Anxiety,” “anxious,” and “panic”

**Fear of recurrences**

“Fear of recurrence” and “recurrence”

**Sleep disturbances**

“Sleep,” “insomnia,” “wake,” “asleep,” “disturb,” “restless,” and “sleep disturbance”

**Coping**

“Coping,” “cope,” and “coped”

**Worry**

“Worry,” “worried,” “worrying,” and “worries”

**Illness intrusiveness**

“lifestyle,” “intruding,” “illness intrusiveness,” “interfere,” “embarrass,” “ashamed,” “shame,” and “disrupt”

**Cognitive changes**

“Fog,” “memory,” “concentrate,” “concentrating,” “concentration,” “cognitive,” “cognition,” and “foggy”

**Educational problems**

“Educational problem,” “student,” “learning difficult,” and “problems in school”

**Social withdrawal**

“Social withdraw,” “withdraw socially,” “social isolation,” “socially isolating,” “lonely,” and “social withdrawal”

**Financial and employment**

“Financial,” “finances,” “employ,” “job,” “fulltime,” “full-time,” “part-time,” and “workload”

**Financial toxicity**

“Financial toxicity,” “debt,” “cost,” “bill,” “expensive,” “expense,” “money,” “money trouble,” and “financial trouble”

**Underemployment, unemployment, and return to work**

“Underemploy,” “unemploy,” “return to work,” “return fulltime,” “return full-time,” “return part-time,” “laid off,” “lay off,” “fire,” “quit,” and “fired”

**Work productivity**

“Work productivity,” “productive at work,” “working hard,” “work hard,” “falling behind,” “fall behind,” and “fell behind”

**School productivity**

“School productivity,” “learning,” and “school college”

**Insurance status**

“Insurance,” “insured,” “Medicaid,” “Medicare,” and “copay”

**Interpersonal**

“Interpersonal,” “boyfriend,” “husband,” “spouse,” “girlfriend,” “wife,” “significant other,” “fiancé,” “partner,” and “relationship”

**Sexuality and intimacy**

“Sex,” “intimacy,” “intimate,” “intercourse,” “sexuality,” and “sexual”

**Fertility**

“Fertility,” “fertile,” “infertility,” “infertile,” “preservation,” “pregnancy,” “pregnant,” “conceiving,” “conceive,” “miscarriage,” “miscarry,” “IVF,” “in vitro,” “oocyte,” “embryo,” “freeze,” “froze,” “egg,” “sperm,” and “frozen”

**Family and caregiver relationships**

“Mother,” “mom,” “father,” “dad,” “sister,” “brother,” “son,” “daughter,” “friend,” “spouse,” “husband,” “wife,” “partner,” “kid,” “child,” “family,” “caregiver,” “relationship,” “friendship,” “partnership,” “marriage,” “divorce,” “separate,” “engage,” and “fiancé”

**Recommended evaluation provided (eg, laboratory testing, imaging, or referral to specialty care)**

“Psychological evaluation,” “social history,” “referral to a therapist,” “referral to a psych,” “referral to psych,” “social work referral,” “referral to social work,” “referred to social work,” “referred to a social work,” and “referred to a psych”

**Treatment provided (eg, medication, therapy, or exercise)**

“Psychological treatment,” “psychological medication,” “counseling,” “therapy,” “support group,” “Zoloft,” “Xanax,” “Lexapro,” “Celexa,” “Wellbutrin,” “Desyrel,” “Prozac,” “Adderall,” “Ativan,” “Cymbalta,” “Effexor,” “Seroquel,” and “Depakote”

**Assessment of adherence to treatment completed**

“Adherence,” “adhere,” “as instructed,” “stick to,” and “stuck with”

**Reassessment of symptoms and conditions at defined intervals or treatment phases**

“Reassessment of psychological symptoms,” “reassess psychological symptoms,” “review psychological symptoms,” and “reviewed psychological symptoms”

### Keyword Verification

To determine whether our keywords were effective, a 2-step verification process was completed on 20 randomly selected posts from the gynecological cancer discussion boards. The goal of the verification process was to (1) check that the computer program appropriately categorized the keywords into their intended themes of interest based on the number of keywords present in the post and (2) verify that an individual naïve to the keywords would categorize the posts as being most related to the same theme that the computer program determined based on which theme had the greatest number of keywords represented. For the first step of keyword verification, a research team member (EA) manually assigned the 20 randomly selected posts to their psychosocial themes using the keyword list and noted which theme had the greatest number of keywords present in the post. EA created the keyword list and was therefore familiar with the keywords. This step was used to verify that the computer program properly captured each theme in a given post based on the number of keywords present for it.

The second step of the keyword verification process was completed by another research team member (MH), who had never seen the keyword list before. MH blindly assigned each of the posts to a theme within the *Surveillance and Management of Psychosocial Effects Domain* without the use of keywords. This step was used to verify that the keywords EA had created for each of the themes within the *Surveillance and Management of Psychosocial Effects Domain* were properly captured.

To determine whether the responses of EA and MH aligned with the computer program’s responses for categorizing the 20 posts into their most relevant theme (the theme with the highest theme score), another research team member (DT) compared the 20 posts’ theme designations by EA, MH, and the computer program. This keyword verification process was successful with a few minor discrepancies that were deemed acceptable as some of the predetermined themes were very closely related (eg, a survivor may be concerned that their “work productivity” could lead to them losing their job and becoming “unemployed,” which are 2 different but strongly related themes). In the few occurrences of a discrepancy between the program and the researchers, the program still scored the posts very highly in the themes the researchers chose, meaning that the post still largely reflected the theme selected by the researchers and the computer program. Thus, a query to find ACS posts discussing themes designated by either the researchers or the computer program would have highlighted the post.

### Applying the Keywords

Once we were confident that our keywords and methodology captured the themes from the *Surveillance and Management of Psychosocial Effects Domain*, we used the methodology at large for all 125,489 posts. To automate the generation of the “theme score” for all posts and all of the predetermined themes of interest, we created another Python script using the base string library. Each post had a theme score calculated for every theme, with the theme score reflecting the number of times the theme’s keywords appeared in the post.

Next, we were able to sort the posts’ theme scores for every theme. This was done so that we could find the most relevant posts within a given theme. Simultaneously, we could see which themes were most prevalent in a given post. For us to consider a post to be “related” to a particular theme, we set a minimum theme score of 3 (a post must contain ≥3 instances of a theme’s keywords). Setting a minimum theme score minimized the number of times someone used a keyword spuriously in a way that was not necessarily relevant to the predetermined theme of interest; for example, a survivor writing “unemployed” once in their post would not prompt the computer program to label it as being related to the “Underemployment, unemployment, return to work” theme.

### Ethics Approval and Data Use

Publicly available data, such as the posts contained on the ACS discussion boards, do not constitute research with human subjects per the Office for Human Research Protections. Thus, institutional review board review approval was not required for the conduction of data analysis, interpretation, and dissemination of findings, as supported by 45CFR46:102 [25]. This is in line with the institutional review board process of all affiliated institutions involved in this analysis.

### Analysis

#### Quantitative Analysis

The process of web scraping and data collection using our methodology generated various quantitative data points to analyze and describe graphically using R and the *ggplot2* package for plotting; for example, we determined which predetermined themes of interest were most frequently discussed on the ovarian, uterine, and gynecological cancers (other than ovarian and uterine) discussion boards. We also examined trends over time across the discussion boards in terms of discussed themes. We determined the prevalence of each theme by taking the number of posts related to a particular theme and dividing it by the total number of posts that year.

Predetermined themes of interest and their respective keywords aside, we wished to pictorially display the words that survivors and caregivers used in their posts. To capture this, we created a graph depicting the 40 most frequently used words in the ACS posts from all 125,498 posts.

#### Qualitative Analysis

By using the keyword list and capturing the most salient predetermined themes in a given post through its theme score, we could easily determine which ACS posts were related to particular themes of interest. This facilitated further qualitative analysis; for example, if we wished to see *how* survivors and caregivers spoke about the theme “Underemployment, unemployment, return to work,” then we could sort through all 125,498 ACS posts to determine which had the greatest theme scores for “Underemployment, unemployment, return to work.” Thus, this would streamline qualitative review such that the select posts related to the theme of interest were already identified and could promptly be analyzed.

To demonstrate how our methodology expedites qualitative analysis in a very large discussion-based data set, we qualitatively analyzed 20 posts. A sample size of 20 was selected based on previously described qualitative research recommendations [26,27]. We selected these 20 posts as they had the absolute greatest number of keyword instances across all of the predetermined themes. Stated another way, for each ACS post, we added up all of the “theme scores” (number of times the keywords were present for a given theme) from all of the themes. The 20 posts that were qualitatively reviewed had the greatest total when adding together all of the individual theme scores. As a number of ACS posters were quite active on the discussion boards, a maximum of 5 posts per user was included in the analysis. If the sixth post from a poster qualified for inclusion based on its theme score, it was instead excluded, and the next post from another unique user was included.

These qualitative analyses were completed by 2 research team members (EA and MH). Each post was individually examined, and the reviewers noted quotes that they deemed to be the most relevant to the themes in the *Surveillance and Management of Psychosocial Effects Domain*. Any discrepancies between the reviewers (EA and MH) that could not be resolved between them were resolved by a third reviewer (ML) to determine the final assigned themes. EA and MH also collected any demographic information available within the ACS posts about the survivor or caregiver who posted.

## Results

### Quantitative Analysis

**Figure 1:**
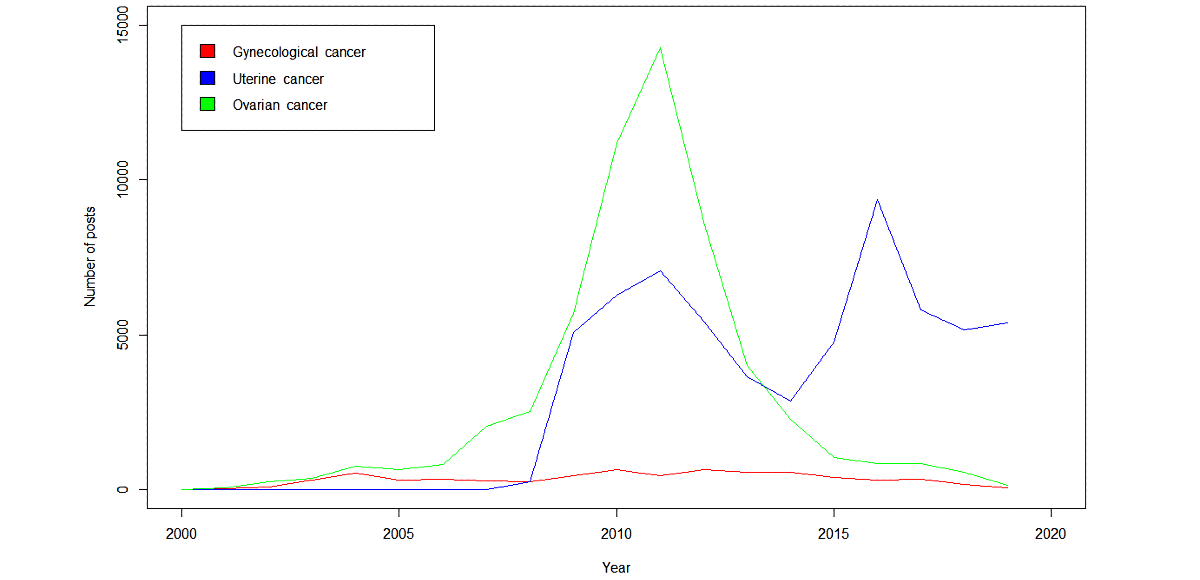
Number of posts by discussion board over time.

A total of 125,498 posts were analyzed quantitatively, with 61,699 (49.16%) posts from the uterine cancer discussion board; 57,011 (45.43%) from the ovarian cancer discussion board; and 6788 (5.41%) from the gynecological cancers (other than ovarian and uterine) discussion board. These posts were created by a total of 6436 unique posters, with each unique poster creating an average of 19.5 (SD. 107.4, range 1-2397) posts over the 20-year period that was evaluated. The total number of posts experienced a large boost in 2008 and reached its maximum in 2011, with the ovarian cancer discussion board being the most prolific. The uterine cancer discussion board experienced a notable rise in posts in 2011 and 2016, and the gynecological cancers (other than ovarian and uterine) discussion board had a steady number of posts over time, as depicted in Figure 1. After this point, the number of posts on the ovarian cancer discussion board markedly decreased, whereas the uterine cancer discussion board maintained its higher number of posts and remained on an upward trend after this point.

**Figure 2:**
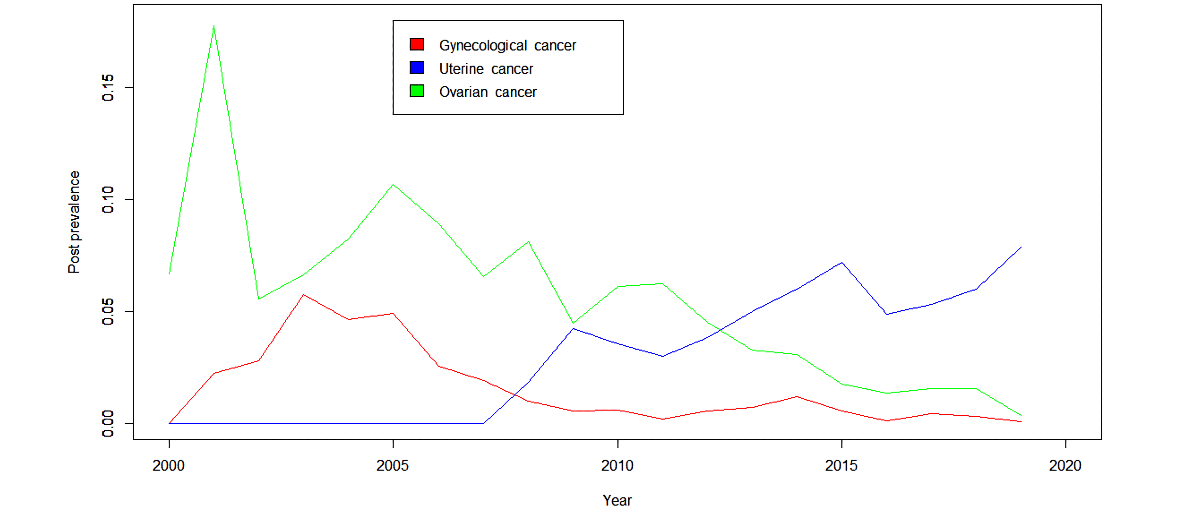
Prevalence of psychosocial themes over time.

Of the 125,489 total posts, 23,458 posts (18.69%) were related to the psychosocial experience of cancer and were further investigated based on the presence of at least a theme score of 3 from the *Surveillance and Management of Psychosocial Effects Domain*. As illustrated in Figure 2, the themes from the *Surveillance and Management of Psychosocial Effects Domain* had become more prevalent within the uterine cancer discussion board over time. The prevalence of these psychosocial themes of interest within the ovarian cancer and gynecological cancers (other than ovarian and uterine) discussion boards had decreased over time; however, this may be due in part to the decreasing number of overall posts within these 2 discussion boards. The most frequently discussed predetermined themes that survivors posted about across all of the gynecological cancer discussion boards were related to (1) the role of friends and family in care, (2) fatigue, (3) the impact of interpersonal relationships, and (4) health insurance status. Additional prevalent themes of interest that were noted were related to stress, sexuality, and fertility.

**Figure 3:**
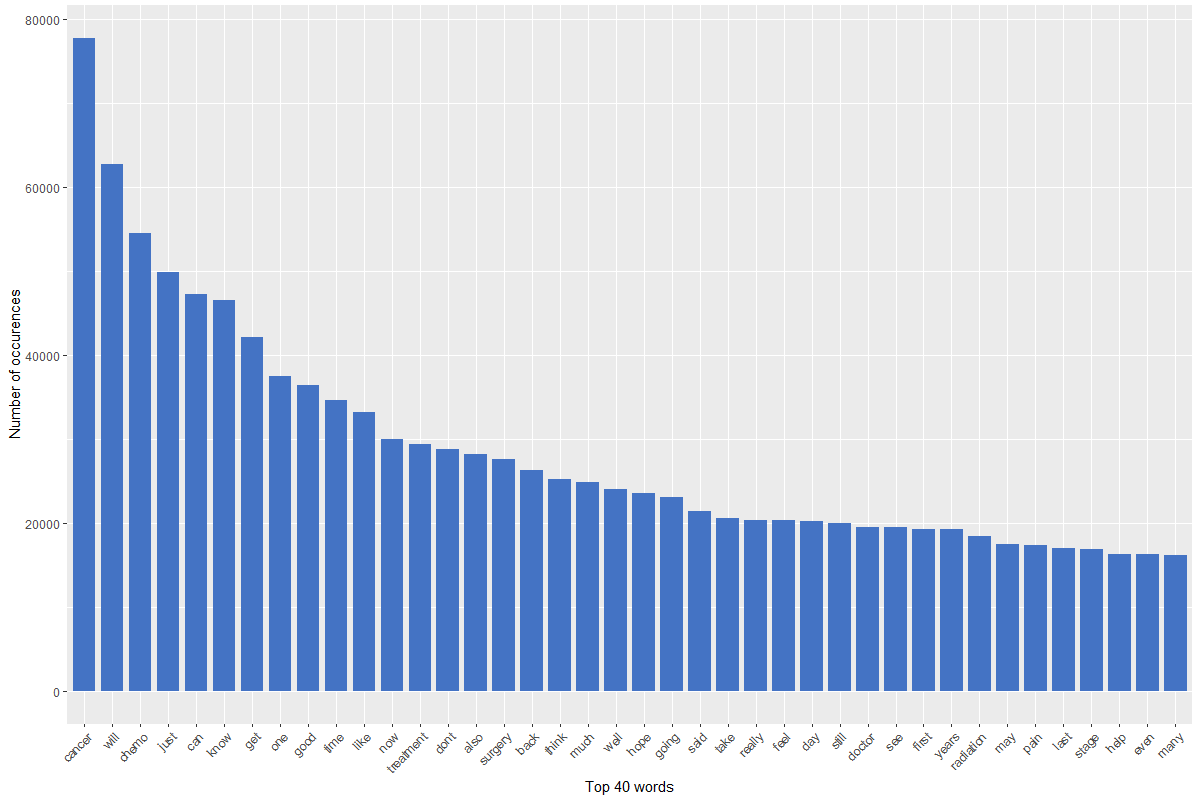
Most frequently used words in posts.

The most prevalent words seen in the posts from all evaluated discussion boards, regardless of their relatedness to the predetermined psychosocial themes, are depicted in a graph (Figure 3). Most words used by survivors and caregivers in the ACS posts pertained to the treatment of cancer, including “cancer,” “chemo,” “treatment,” “surgery,” and “radiation.” Many words were used by survivors that were related to survivorship based on psychosocial themes, including “family,” “hope,” “help,” and “pain.”

### Qualitative Analysis

The 20 posts included in our proof-of-concept qualitative analysis included 238 quotes that were determined to be of interest. These 20 posts came from 8 different users who were either survivors or caregivers of patients with ovarian or uterine cancer. Of the 20 posts, most concerns that were related to the predetermined themes of interest in the *Surveillance and Management of Psychosocial Effects Domain* were related to coping with distress and worry, family or caregiver relationships, and the financial hardships of cancer. Textbox 2 includes representative quotes that discuss these highlighted themes.

In exploring the qualitative aspects of the observed psychosocial posts, additional nuances came to light. A notable amount of the discussion that was related to the psychosocial themes of distress and worry was related to concerns regarding the disease returning, progressing, or metastasizing. This gave us insight into the theme of coping as well, where we observed two salient approaches: taking an active role in care and the role of spirituality. Coping by taking an active role in care was evident as posters requested opinions on therapeutic options, shared and interpreted primary literature with one another, asked for help devising questions for their physician, and brainstormed strategies to self-advocate. A distinct element of this was encouraging others to seek a second opinion, as demonstrated by a uterine cancer discussion board poster:

A second opinion may prove lifesaving for you. There are options out there for you in spite of this disappointing news, so don’t let a busy doctor write you off. Don’t write yourself off either. If you have to go down, go down fighting. Do it for yourself, do it for your husband, and do it for those dreams you have.

In addition to augmenting self-efficacy, another component of coping was spirituality. Many shared the way that faith provided them reassurance in adjusting to a terminal diagnosis and accepting one’s mortality. A patient with metastatic ovarian cancer shared the following:

Contrary to what others may choose to believe, while I know that God can and does heal many, He isn’t obligated to do so. And I’m not here to challenge anyone else’s beliefs, just telling you where I come from as it relates to my own Stage IV diagnosis.

Survivors also frequently discussed the relationships they had with family and caregivers, with 1 important element being focused on helping loved ones adjust emotionally to the patient’s cancer diagnosis, treatment, and prognosis. A survivor of ovarian cancer created a post on how spouses, parents, adult children, friends, and colleagues may support a loved one with cancer, which included (1) rallying the support network to delegate tasks and increase overall support, (2) remembering to take care of oneself so that they are better able to support the patient, and (3) attending support groups for caregivers. Another striking post was from the husband of a woman with ovarian cancer who asked for advice on how to speak to his wife about her cancer and prognosis while she anecdotally was in denial. A survivor replied, suggesting the following:

tell her you’re researching her cancer. If she asks more, tell her more. If she wants to live in denial, that’s her choice...if she becomes curious enough to ask more, that could be an opening for a deeper conversation. Ideally the two of you need to be able to face the truth together. She will be the one suffering the physical and emotional aspects of this diagnosis. You will certainly be suffering emotionally...and believe me, she wants to live as much as you want her to live...As a caregiver, I know your heart aches.

Another theme that was prevalent and provided interesting insights into the lived experience of survivorship was the intersection of financial toxicity and insurance status. Conversations were particularly focused on accessing treatment options. A survivor of uterine cancer posted the following:

During all this I’m going though, now my insurance is denying paying for the Avastin. I have no idea how much it would cost out of pocket.

Another survivor replied with the following advice:

They will just deny almost as a matter of course unless they’re disputed. People are denied all the time and get it overturned after some dispute...They start off with denial and then see if the doctor really means it or not...You really really really have to be on your doctor’s office to find out exactly what was submitted, exactly what your insurance requires and exactly why it was denied, and getting your doctor to fight. You shouldn’t have to...but you really really really do.

This provided insights into the experiences that patients may be facing, which affect their quality of life outside of cancer itself.

As demonstrated, the qualitative analysis provided insights into *how* survivors discussed particular themes of interest and gave insights into how these themes are related to one another. Notably, the vast majority of the analyzed quotes from the 20 posts touched on >1 theme in the *Surveillance and Management of Psychosocial Effects Domain*. The least commonly observed psychosocial survivorship themes of interest were related to adherence to treatment, educational and occupational hardships, and social withdrawal.

**Quotes from the qualitative analysis.**

**Themes and quotes from discussion board posts**

**Coping**

“I’m 42. I weep for the years I’m likley to lose to this cancer and at the rate it’s growing I fear I only have months now.”

**Distress and worry**

“I speak as a Stage IV cancer patient, and know the effects I have already suffered through, and I’m not about to ‘try one more’ for only 3 more months of survival, which would not be absent side effects which are many, during that time. I speak only for myself, but my mind is made up!”

**Psychological aspect of physical effects**

“Often it’s quite debilitating. And we may even despair at times when side effects seem intolerable.”

**Insurance status and financial toxicity**

“Boy what a pain in the butt. I’m sorry you have to fight this money stuff. You have enough to worry about. Boy don’t they know how urgent and important this is. At the same time this just shows what a great fighter you are. I really hope you get this cleared so you can get the treatments you need.”

**Family and caregiver relationships**

“[as a caregiver], allow them to vent, don’t take it personally, know it’s the confusion and disease that's talking and all of their fears...Be strong. Don’t borrow trouble, but be realistic to the prognosis and day-by-day plan, attitude is huge in the battle and living in the now and positivity is key”

### Topic Modeling

Although LDA was able to separate the posts into different themes, the resulting themes were broad. Investigating a model that discovered 16 rather than 8 themes led to more specific topics, although many were deemed random and irrelevant; therefore, analysis of the 8-theme model was continued. The 8 themes that LDA discovered from greatest theme prevalence to least theme prevalence were support, treatment side effects, diagnosis, research and clinical trials, treatment, ovarian cancer, help, and time. These findings are summarized in Multimedia Appendix 1, as well as the keywords the topic modeling paired together for each theme. Notably, there was an overlap of 2 keywords among the LDA-generated keywords and the keywords developed using our primary mixed methods methodology: *sleep* within the “treatment side effects” discovered theme and *work* within the “time” discovered theme.

## Discussion

### Principal Findings

This study was conducted to apply a novel methodology that was developed to examine a large web-based, narrative-based data set using mixed qualitative and quantitative methods. This methodology offers both the ability to describe and leverage extremely large quantities of data using quantitative techniques while simultaneously guiding and streamlining qualitative analysis. We demonstrated the utility of this methodology in the context of the previously published Quality of Cancer Survivorship Care Framework, with a focus on the framework’s *Surveillance and Management of Psychosocial Effects Domain*. We were able to observe both the number of posts made per year across each discussion board, as well as the trends in particular themes of interest across time since the discussion boards’ inception. This provided us with a broad overview of engagement on the platform. Quantitative analysis revealed that survivors frequently discussed the role of friends and family in care, as well as fatigue, the impact of interpersonal relationships, and health insurance status. The most frequently used words that survivors wrote in their posts were related to diagnosis and treatment. The most frequently used words related to the themes that we were interested in from the framework’s *Surveillance and Management of Psychosocial Effects Domain* were “family,” “hope,” and “help.” Qualitative analysis also demonstrated that survivors frequently discussed coping, worry, distress, and family and caregiver relationships, as well as insurance and financial aspects of care. The qualitative analysis provided deeper insights into *how* survivors were affected by these themes and provided greater insights into the nuances of survivors’ unmet concerns.

### Comparison With Prior Work

Social media and discussion-based platforms can provide invaluable information from people affected by cancer and other health conditions; however, the magnitude of data available in social media posts poses a barrier to its use in research [11,28]. Although there are other automated methods to thematically categorize large quantities of written text data in addition to those we have presented in this paper, such as topic modeling [16,17], they often use computer software to generate and cluster themes that the software deems to be of importance. Unfortunately, as we experienced in our analysis, these themes are often not meaningfully relevant enough to answer a specific research question or explore known themes. As we desired to explore the psychosocial aspects of survivorship, the themes that LDA discovered were not specific enough to meaningfully inform our research question. To that end, our methodology ensures that themes of interest are captured while still being an automated process. Another approach to thematically analyzing large quantities of text-based data, which ensures that themes of interest are accurately captured, involves manually extracting data by hand [11-13,15]. However, manual abstraction is often not feasible when wishing to analyze the entirety of the great magnitudes of data that are available on web-based platforms. The methodology we present in this report offers the efficiency of automated thematic analysis while retaining the accuracy and thoughtfulness of manual data abstraction.

The methodology presented in this report may be used to provide clinicians and researchers with invaluable insights, opinions, and suggestions made by survivors of cancer themselves in an efficient and low-cost manner. The candid conversations among survivors publicly available on discussion boards and social media platforms may inform future survivorship efforts and programs, as they reveal the honest and spontaneous concerns, attitudes, and preferences of survivors. This report contributes to the growing body of knowledge extracted from the ACS discussion boards, including a prior publication from our group [15,29]. Additional articles support the richness of the opportunity to explore the perspectives of survivors of cancer through ACS discussion board posts, as demonstrated by Fallon et al [30], who found that 25% of ACS Cancer Survivors Network users return to the site at least monthly, with most interacting with the discussion boards. Harnessing the candid words of survivors will provide survivorship initiatives with an understanding of what is most important to survivors and could perhaps inform clinicians of what is currently missing in survivorship care.

Prior work has explored the unique survivorship needs of those with gynecological cancers. DeRooij et al [31] explored the unmet survivorship needs of patients with gynecological cancers from the perspective of patients, caregivers, and health care providers using semistructured interviews. Almost all participants wished to receive more resources on the side effects of treatment, the anticipated follow-up plan, and psychosocial support. Our analysis contributes to the understanding of what psychosocial supports could be most useful to survivors of gynecological cancer and their caregivers. In fact, Beesley et al [32] distributed a survey to determine the number of survivors of gynecological cancer who had unmet survivorship needs and found that 43% did, with most of their concerns being related to the psychosocial aspects of survivorship. The most common unmet needs included fear of cancer progressing, helping friends and family adjust to their cancer diagnosis, future unknowns, fatigue, and being unable to perform the tasks and activities they enjoyed previously [32]. These findings are very similar to ours, particularly as we found that the greatest worries that survivors discussed on the ACS discussion boards were related to fear of their cancer progressing, metastasizing, or returning. Moreover, the concerns regarding how cancer affects loved ones were salient as multiple posts were related to giving advice on how to help caregivers adjust to their loved one’s diagnosis or how caregivers can best support their loved ones with cancer. Together, our work complements and further informs the available literature on the unmet psychosocial needs of survivors of gynecological cancer.

### Limitations

Although our analysis is strengthened by the data set that included 20 years of posts from the ACS discussion boards, we acknowledge that the cancer care and survivorship needs of patients have greatly changed over this time. Thus, the findings must be independently evaluated to determine how they may be applied to current and future survivorship initiatives. As the qualitative analysis served as a proof of concept, we wish to emphasize that the qualitative analysis of this project is not a comprehensive analysis of all of the posts; rather, it demonstrates how this technique can be used to discuss *how* particular topics are discussed. Thus, the quotes explored in the qualitative analysis may not be reflective of all posts in the data set. Moreover, we acknowledge the limited diversity in the authors of the 20 posts qualitatively analyzed.

### Conclusions

Internet-based discussions among survivors of gynecological cancer offer valuable insights into the unmet psychosocial survivorship needs that can be addressed in future survivorship initiatives. Most often, survivors of gynecological cancer discussed the role of friends and family in care, as well as fatigue, the effect of cancer on interpersonal relationships, and health insurance status, as discovered through the quantitative phase of the analysis. The complementary qualitative analysis informed how these themes affect survivors, showing the specific gaps in survivorship care that may be addressed. This informative and customizable methodology may continue to be applied across clinical settings and patient populations, harnessing the abundance of patient-generated and patient-centered internet data for empirical inquiry.

## Appendix

Multimedia Appendix 1 Themes and keywords discovered via latent Dirichlet allocation.

## Abbreviations

ACS: American Cancer Society
CV: metric of coherence
LDA: latent Dirichlet allocation
