# Supplementary figures and images for: Psychosocial Needs of Gynecological Cancer Survivors: Mixed Methods Study
Source: J Med Internet Res. 2022 Sep 20;24(9):e37757. doi: 10.2196/37757 (PMC9533206; doi:10.2196/37757)

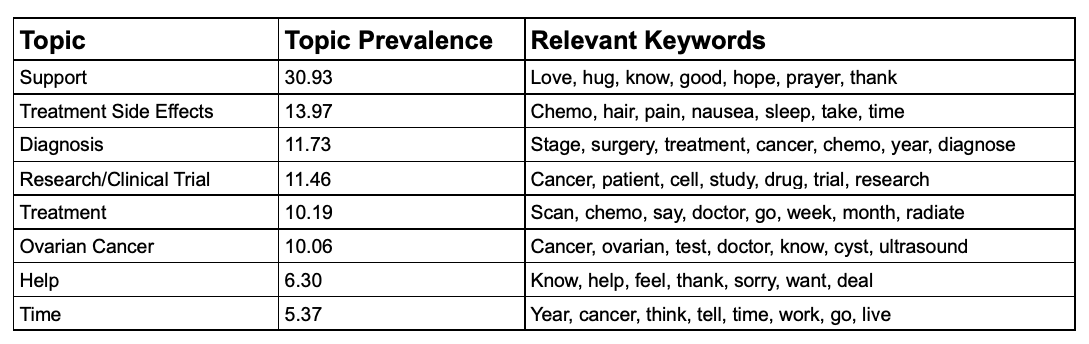

Supplement: Multimedia Appendix 1 [file jmir_v24i9e37757_app1.png]
